# A case of giant panda ovarian cancer diagnosis and histopathology

**DOI:** 10.1186/s12917-018-1630-x

**Published:** 2018-10-12

**Authors:** Qi Gao, Chengdong Wang, Desheng Li, Hemin Zhang, Linhua Deng, Caiwu Li, Zhengli Chen

**Affiliations:** 1 Laboratory of Animal Disease Model, College of Veterinary Medicine, Sichuan Agricultural University, Chengdu, China; 2 China Giant Panda Conservation Research Center, Dujiangyan, China

**Keywords:** Giant panda, Ovarian cancer, Histopathology, Diagnostics

## Abstract

**Background:**

Ovarian cancer is diagnosed clinically by detecting ovarian cancer-related factors and markers. Here, we report a case of giant panda ovarian tumor metastasis with a combination of clinical and histopathological diagnosis.

**Case presentation:**

Histopathological studies revealed severe lesions and tumor cells in the ovaries, lungs, spleen, kidneys and perianal tissue. Immunohistochemistry staining showed that the ovarian cancer markers B7-H4, CA125, and HE4 were highly expressed in the lungs, kidneys, spleen, ovaries and perianal tissue. Tumor marker tests detected significantly high levels of AFP in serum.

**Conclusion:**

Clinical biomarkers combined with histopathology can provide a more accurate diagnosis of ovarian cancer metastasis and identification of ovarian cancer types than either method alone. The giant panda’s death may be due to granulosa cell tumor and theca cell tumor metastasis causing multiple organ dysfunction or even failure.

## Background

Ovarian cancer is the fifth most common cause of cancer death in women behind lung cancer, breast cancer, colorectal cancer, and pancreatic cancer [1]. There are two major types of ovarian cancer: epithelial and germ cell neoplasms. About 90% of ovarian cancers are epithelial and develop on the surface of the ovary. These tumors are often bulky and involve both ovaries. Germ cell tumors are derived from the eggs within the ovary and, if malignant, tend to be highly aggressive [2].

The CA125 antigen is a high molecular weight glycoprotein that is expressed in most epithelial ovarian cancers. It is currently the favored tumor marker for epithelial ovarian cancer and has played an important role in diagnosis and incorporating the risk of a malignancy index. However, the sensitivity and specificity of CA125 is poor, and CA125 is also a valid marker for many diseases other than ovarian cancer [3].

Human epididymis protein 4 (HE4) is a glycoprotein that is highly expressed in ovarian cancer. It is mainly expressed in the reproductive system and in respiratory and salivary gland secretions [4]. It has been proposed as a potential biomarker for ovarian cancer because the combination of HE4 and CA125 was more accurate than any other markers in predicting malignant ovarian tumors [5]. The Food and Drug Administration (FDA) in U.S. has approved HE4 for monitoring the recurrence of disease in ovarian cancer patients [6]. B7-H4, a newly identified member of the B7 family, is involved in the negative regulation of T cell-mediated immunity in peripheral tissues. Research has found that B7-H4 inhibits apoptosis and promotes tumor cell growth, adhesion, and invasion into immunodeficient systems [7]. B7-H4 is overexpressed in ovarian cancer tissues and elevated levels are detected in the serum of ovarian cancer patients, but not in patients with benign diseases [8]. So B7-H4 may play a more important role in tumorigenesis and metastasis. Ovarian cancer is generally diagnosed clinically by detecting ovarian cancer-related factors and markers, but the biomarker analysis alone is not sensitive enough, because the expression of related biomarker in serum can not directly reflect the extent of lesions in the tissue. So here, we report a case of giant panda ovarian tumor metastasis by combination of clinical and histopathological diagnosis. It allows us to visually understand the pathological changes in the tissue and to further diagnose the disease by detecting biomarkers.

## Material and methods

### Histopathology

The body of the giant panda was systematically dissected. Specimens from the heart, lungs, liver, spleen, kidneys, perianal tissue, and ovaries were fixed in 4% paraformaldehyde phosphate buffer and embedded in paraffin using routine protocols. Paraffin-embedded materials were sectioned at 5 μm for staining with H&E (hematoxylin-eosin).

### Immunohistochemistry

Sections were dewaxed in xylene and dehydrated in alcohol and antigen retrieval was achieved by microwaving them in citrate solution for 15 min. They were then soaked in 0.3% hydrogen peroxide for 20 min to block endogenous peroxidase activity. Sections were blocked by 5% bovine serum albumin and incubated with the primary antibody against CA125 (Boiss, China), HE4 (Boiss, China) and B7-H4 (Boiss, China) at 4 °C overnight. After rinsing, a rabbit IgG SABC (StreptAvidin-Biotin Complex) immunohistochemical staining kit (Boster, China) was used according to the manufacturer’s protocols. Tissue sections were visualized with the substrate DAB (diaminobenzidine) chromogen (Boster, China). Positive results were dark blue particles, located in the cytoplasm or nucleus.

## Case presentation

**Fig. 1:**
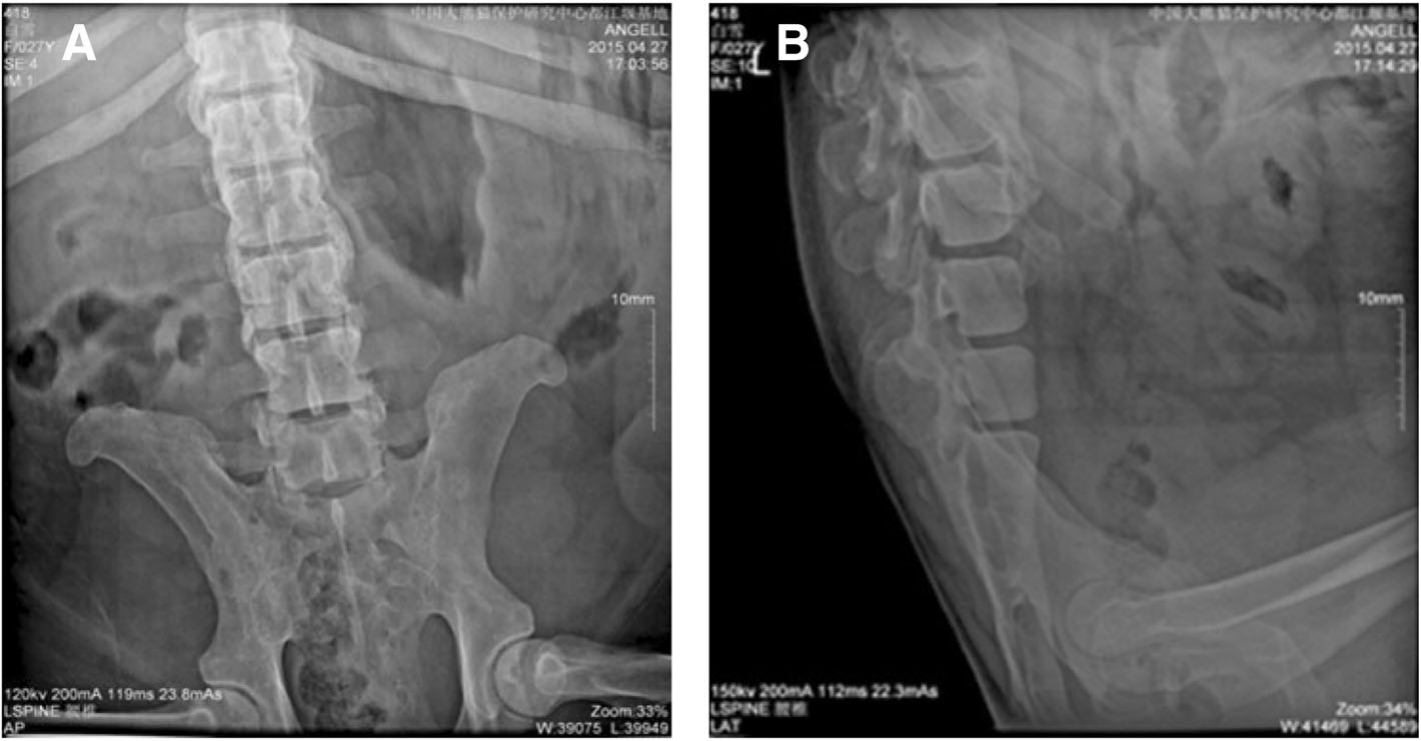
The results of Digital Radiography diagnosis. The lateral longitudinal ligament and anterior longitudinal ligament of the lumbar occurred band calcification with the formation of a bridge of the bone. **a** Lumbar anteroposterior position. **b** Lumbar lateral position

**Fig. 2:**
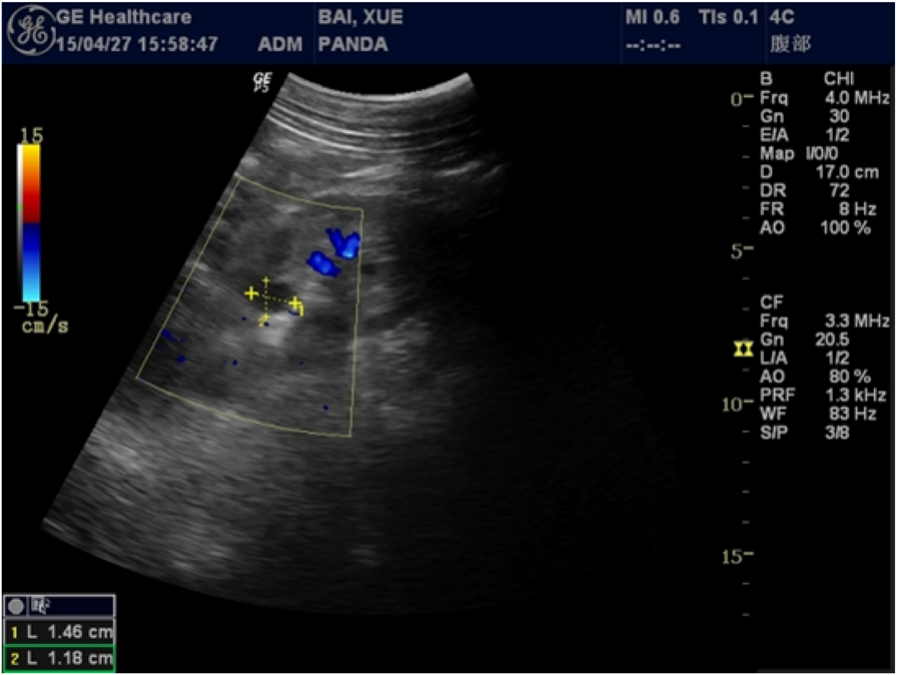
The results of B-scan ultrasound. We found a 1.46 × 1.18 cm cyst in the left kidney. Blue indicates blood flow away from the probe

The giant panda was a female who was rescued in the wild in Shaanxi Province, China in 1988. The pedigree number is 418. She started to reduce her activity, walking slowly, and her activity did not increase during estrus since March 9, 2015. On April 7, she was found to walk more slowly. Her hindlimbs were unable to support her as she walked, and the main manifestations were ankylosis, lying or standing eating, and the inability to curl up or sit on April 11. We suspected that her waist was injured and conducted a systemic examination after anesthesia on April 27, 2015, including basic physiological indicators tests, routine blood tests, blood biochemical tests, a DR (Digital Radiography) test, and a B-scan ultrasound test. The results showed that the number of white blood cells and AST / ALT ratio was elevated (Tables 1 and 2), suggesting that there was inflammation in her body. Furthermore, the lateral longitudinal ligament and anterior longitudinal ligament of the 10th thoracic vertebra and the third lumbar showed band calcification with the formation of a bridge of the bone (Fig. 1). A 1.46 × 1.18 cm cyst was found in the left kidney by B-scan ultrasound (Fig. 2). After taking Cefalexin 2 g/times and special nursing (daily massage for waist, hot compress) for 7 consecutive days, the patient’s condition further deteriorated and she was lying sideways in the house with almost no ambulation. Hematological tumor marker tests revealed a significant increase in AFP (alpha-fetoprotein) values (Table 3). Eventually she died at 18:00 on June 12, 2015.

**Table 1:** The results of routine blood examination

Projects	Value	Unit
WBC	15.61	10E9/L
Neu%	86.9	%
Lym%	6.1	%
Mon%	2.6	%
Eos%	4.3	%
Bas%	0.10	%
Neu#	13.56	10E9/L
Lym#	0.95	10E9/L
Mon#	0.41	10E9/L
Eos#	0.67	10E9/L
Bas#	0.02	10E9/L
RBC	7.15	10E12/L
HGB	131	g/L
HCT	36.50	%
MCV	51.1	fL
MCH	18.3	pg
MCHC	359	g/L
RDW-CV	14.4	%
RDW-SD	32.3	fL
PLT	1470.0	10E9/L
MPV	6.6	fL
PDW	15.3	f1
PCT	0.970	%

**Table 2:** The results of blood biochemical examination

Projects	Value	Unit
TBIL	6.7	μmol/L
TP	62.0	g/L
ALB	21.5	g/L
AST	101.0	U/L
ALT	25.4	U/L
ST/LT	3.98	/
PA	57.4	mg/L
CHE	546	U/L
TBA	22.20	μmol/L
Cr	71.5	μmol/L
UA	144.0	μmol/L
TG	5.68	mmol/L
CHOL	6.70	mmol/L
HDL	2.13	mmol/L
LDL	3.26	mmol/L
APOA1	0.25	g/L
APOB	0.09	g/L
GLU	2.36	mmol/L

**Table 3:** The test results of tumor markers in serum

Projects	Value	Unit
AFP	70.4	ng/ml
CA125	10.00	U/ml
CA15–3	1.20	U/ml
CA19–9	10.50	U/ml
CEA	5.00	ng/ml

**Fig. 3:**
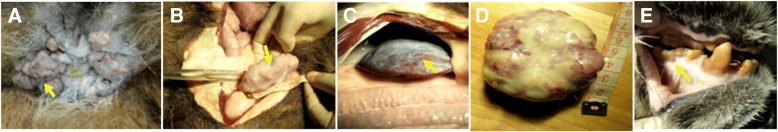
Anatomical pathology of the main organ lesions. **a** Many neoplasms in the perianal area. **b** Ovarian tumor. **c** A large number of ovarian neoplasm. **d** Hard texture of the liver, fat accumulation on the surface. **e** Visible ptosis in the left upper part of the gums

The corpse weighed 85 kg and had more than a dozen neoplasms in the perianal area (Fig. 3a). Anatomical examination found a large number of ovarian neoplasms, the largest at 7 cm in diameter (Fig. 3b). The liver was hard in texture and had fat accumulation on the surface (Fig. 3c). The lungs were also hard and inelastic. There were visible ptosis in the left upper part of the gums (Fig. 3d) and a total of about 5000 ml in ascites.

**Fig. 4:**
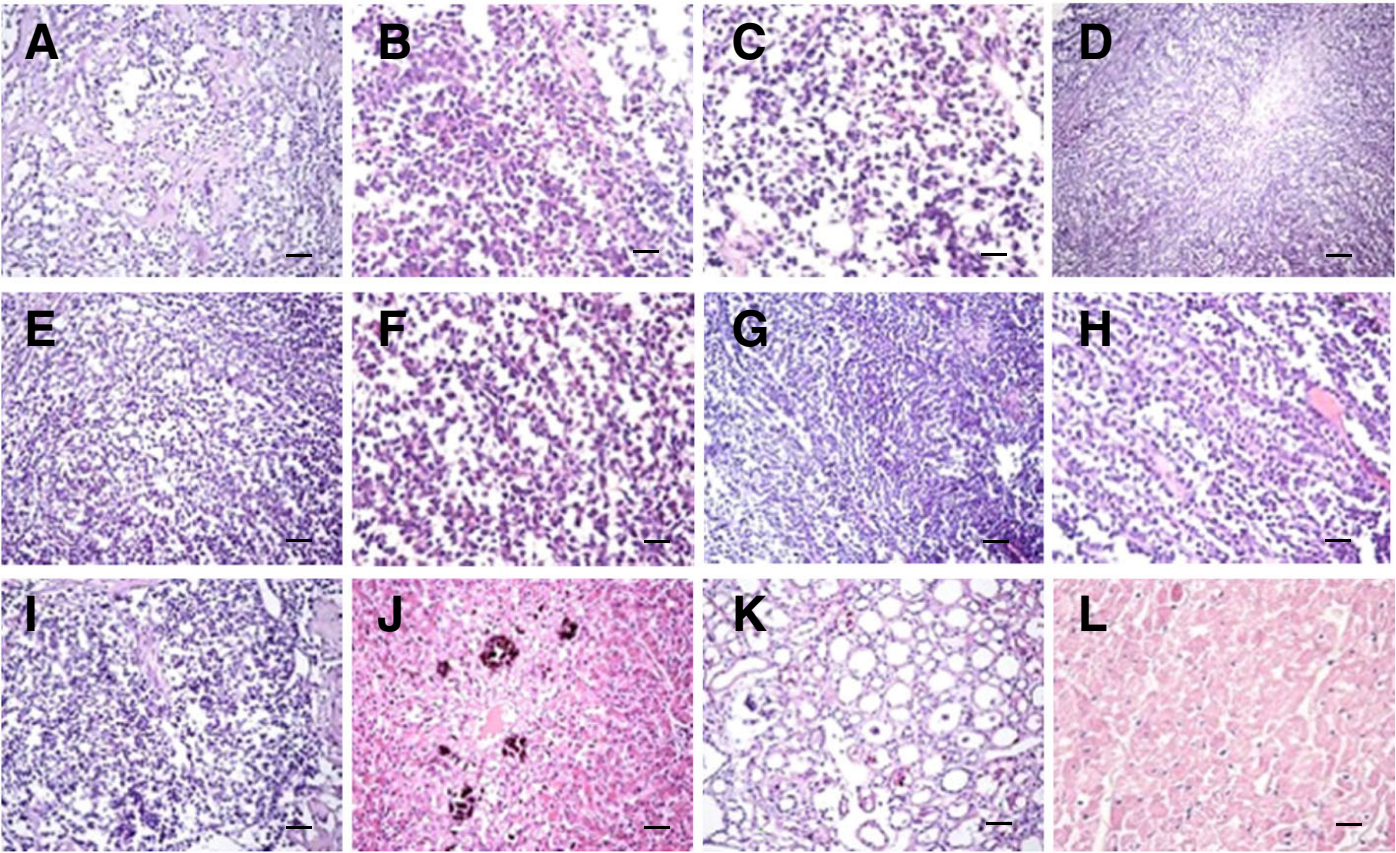
Histopathological results of main lesion. **a-c** Severe alveolar atrophy or even disappearance, pulmonary fibrosis hyperplasia in the lung, visible tumor cell metastasis. **d-f** Ovarian tissue showed a large number of round cells were nests or cord-like proliferation, spindle-shaped cells proliferation in the periphery, accompanied by the formation of collagen fibers, tumor cell atypia is not obvious, rare cells split phase, local necrotic calcification area. **g-h** Perianal tissue showed tumor cell metastasis accompanied by fibrogenesis. **i** Fewer spleen lymphocytes than normal, monocyte proliferation. **j** Severe hepatic steatosis, partial necrosis, multifocal calcification, small focal inflammatory infiltration of the liver. **k** Part of glomerular atrophy, mild glomerular swelling, tubular significant dilatation, visible proteinuria and cell tube in the kidney. **l** Severe swelling of myocardial cells, granular degeneration with partial necrosis in the heart. (**d**: Bar = 100 μm. **a**, **e**, **g**, **i**, **j** and **k**: Bar = 50 μm. **b**, **c**, **f**, **h** and **l**: Bar = 20 μm)

Histological examination showed severe alveolar atrophy or even disappearance, pulmonary fibrosis hyperplasia in the lungs, and visible tumor cell metastasis (Fig. 4a-c). Ovarian tissue showed a large number of round cells were nests or cord-like proliferation, spindle-shaped cells proliferation in its periphery, accompanied by the formation of collagen fibers, tumor cell atypia is not obvious, rare cells split phase, and local necrotic calcification area (Fig. 4d-f). Perianal tissue showed tumor cell metastasis accompanied by fibrogenesis (Fig. 4g-h). There were fewer spleen lymphocytes than in normal, and a proliferation of monocytes (Fig. 4i). In the liver we saw severe hepatic steatosis, partial necrosis, multifocal calcification, and small focal inflammatory infiltration (Fig. 4j). In the kidneys we saw partial of glomerular atrophy, mild glomerular swelling, tubular significant dilatation, visible proteinuria, and cell tubes (Fig. 4k). In the heart we saw severe swelling of myocardial cells and granular degeneration with partial necrosis in the heart (Fig. 4l).

**Fig. 5:**
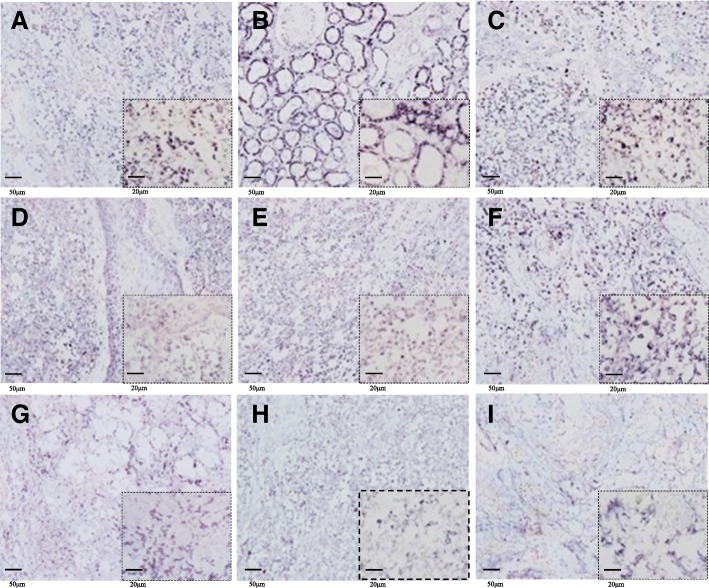
Immunohistochemistry for B7-H4, CA125 and HE4. **a-c** B7-H4 is abundantly expressed in the cell membranes of the lungs, kidneys, and spleen. **d-f** CA125 is abundantly expressed in the cell membranes and cancer cells of perianal tissue, ovaries, and lungs. **g-i** HE4 is highly expressed in cancer cells of the lungs, ovaries, and spleen

Immunohistochemical staining showed that the ovarian cancer markers B7-H4, CA125, and HE4 were highly expressed in the lungs, kidneys, spleen, ovaries and perianal tissue (Fig. 5).

## Discussion and conclusions

Blood tests showed inflammation in the body of the giant panda, and we treated her with anti-inflammatory treatment. The giant panda was found with ligament calcification and formation of bone bridge by DR examination. Combined with her daily performance, we diagnosed her as senile with degenerative spondylitis [9]. There is still a lack of treatment for this disease in current medical research; we could only minimize her pain with methods like massage.

Severe lesions in multiple organs were observed by histopathology. Moreover, the visible ovarian granulosa cells and theca cells become cancerous in the ovary and even transferred to the lungs, kidneys, perianal tissue and spleen. This may have severely affected the functioning of these organs. We also detected high expression of B7-HE, CA125, and HE4 by immunohistochemistry in the lungs, ovaries, kidneys, perianal tissues, and spleen. The metastasis of ovarian tumors to these areas was confirmed by the detection of ovarian cancer biomarkers in these tissues [10]. CA125 and overexpressed AFP were also detected in serum. AFP is a tumor antigen that is expressed in cells during embryonic development and is abundantly expressed in adult tumor cells. It is useful in the clinical detection of tumor markers [11]. There is research showing that malignant ovarian tumors include germ cells and sex cord stromal tumors, which are associated with marked elevation serum of AFP [12, 13].

Ovarian granulosa cell tumors (GCTs) are uncommon neoplasms that arise from the sex-cord stromal cells of the ovary [14]. It has been reported that metastasis of ovarian granulosa tumor to the lung leads to structural changes and dysfunction in the lung tissue [15]. This giant panda is diagnosed with ovarian cancer [16]; her death may be due to granulosa cell tumor and theca cell tumor metastasis leading to multiple organs dysfunction or even failure. In summary, clinical biomarkers combined with histopathology can provide a more accurate diagnosis of ovarian cancer metastasis and identification of ovarian cancer types. In clinical examination, in addition to detecting biomarkers of ovarian cancer in serum, it is possible to perform histopathological examination by biopsy.
